# Exploring Adult Eating Behaviors and Food Neophobia: A National Study in Romania

**DOI:** 10.3390/foods13091301

**Published:** 2024-04-24

**Authors:** Meda-Ada Bugi, Iulius Jugănaru, Iulia-Elena Simina, Delia-Maria Nicoară, Lucian-Ioan Cristun, Giorgiana-Flavia Brad, Casiana Boru, Daniela Cîrnatu, Otilia Mărginean

**Affiliations:** 1 Ph.D. School Department, ‘Victor Babeş’ University of Medicine and Pharmacy of Timisoara, 300041 Timisoara, Romania; bugi.ada@umft.ro (M.-A.B.); nicoara.delia@umft.ro (D.-M.N.); lucian.cristun@umft.ro (L.-I.C.); 2 Department of Pediatrics I, Children’s Emergency Hospital ‘Louis Turcanu’, 300011 Timisoara, Romania; juganaru.iulius@umft.ro (I.J.); brad.giorgiana@umft.ro (G.-F.B.); marginean.otilia@umft.ro (O.M.); 3 Department of Pharmacy, University of Medicine and Pharmacy ‘Vasile Goldis’, 310025 Arad, Romania; 4 Department XI Pediatrics, Discipline I Pediatrics, ‘Victor Babeş’ University of Medicine and Pharmacy of Timisoara, 300041 Timisoara, Romania; 5 Department of Research Center for Disturbances of Growth and Development in Children–BELIVE, ‘Victor Babeş’ University of Medicine and Pharmacy of Timisoara, 300011 Timisoara, Romania; 6 Department of Genetics, Center of Genomic Medicine, ‘Victor Babeş’ University of Medicine and Pharmacy, 300041 Timisoara, Romania; simina.iulia@umft.ro; 7 Department of Medicine, University of Medicine and Pharmacy ‘Vasile Goldis’, 310025 Arad, Romania; boru.casiana@uvvg.ro; 8 Romanian National Institute of Public Health, Regional Centre, 300230 Timisoara, Romania

**Keywords:** food neophobia scale translation, food disorders, public health, epidemiology

## Abstract

Food neophobia involves avoiding new foods due to reluctance, aversion, or disgust. The Food Neophobia Scale (FNS) is the most reliable and common adult food neophobia test. It helps compare food neophobia across cultures by being translated into numerous languages. This study adapted, translated, and validated the FNS for Romania. This translated version was piloted in November 2023 on 59 students in the medical field from two distinct Romanian cities. Between December 2023 and February 2024, 375 adults were surveyed, representing Romania’s population within a 90% confidence interval. The average age of responders was 38.07 years, with a standard deviation of 10.75 and a 4:1 female-to-male sex ratio. The Cronbach’s alpha test was used to validate the questionnaire. Our study found that the mean FNS value was 31.86; most Romanian respondents (69.20%) were neutral towards trying new foods, with a significant portion being neophobes (18.21%) outnumbering neophiles (12.59%). When compared to international study results, Romanian adults, on average, exhibited a higher percentage of neophobes compared to those in Korea (13%), Hungary (16.8%), Italy (17%), and Brazil (17.5%), but a lower percentage than those in Lebanon (21.5%). The findings indicate that the translated scale can be utilized to assess neophobia among Romanian speakers.

## 1. Introduction

Food neophobia involves a reluctance, aversion, or distaste toward novel foods, potentially resulting in the avoidance of unfamiliar items. This predisposition is shaped by a complex interplay of factors, including genetic predisposition, personal experiences, environmental influences, media and marketing messages, educational background, and cultural traditions. Collectively, these elements influence an individual’s openness to specific foods over time [1,2]. It is important to differentiate neophobia from idiosyncratic food preferences or aversion to unfamiliar dishes within one’s community [3]. This behavioral trait, commonly observed in children, can persist into adulthood due to a variety of environmental influences, including cultural food practices, socio-demographic characteristics, lifestyle, educational background, and the aging process [2,4,5,6,7]. These diverse elements collectively influence an individual’s tendency to be averse or hesitant towards unfamiliar foods. 

Therefore, individuals exposed to diverse cultures may exhibit lower levels of food neophobia [5]. The willingness to try new foods is also influenced by an individual’s knowledge and personal experiences [8]. When examining social factors, culture was found to influence neophobia [9,10,11]. Individuals who engage in frequent travel and display openness to the cultures of other countries generally exhibit reduced neophobic behavior [2,9,12]. The advent of cultural diversity and globalization has introduced a plethora of novel food items alongside traditional options in the market. Consequently, the examination of neophobia has resurfaced as a pertinent and timely issue [13,14,15]. Psychological factors are also associated with the concept of “familiarity” [16], which must be carefully defined in the context of neophobia [17].

One such tool is the Food Neophobia Scale (FNS), which evaluates individuals’ hesitancy or resistance toward trying new or unfamiliar foods. Additional instruments are utilized, including the Children’s Eating Behavior Questionnaire (CEBQ), Fruit and Vegetable Neophobia Inventory (FVNI), Food and Eating Questionnaire (FEQ), and others, all falling under the category of Questionnaires and Indices in Instruments to Measure Neophobia and Willingness to Try Unfamiliar Food [18].

The FNS was originally developed in English by Pliner and Hobden [8], consisting of a self-administered questionnaire comprising 10 items. For a multinational use, it has been translated and documented in various languages according to the literature, including Swedish [13], Finnish [19], Spanish [20], European Portuguese [21], Brazilian Portuguese [16], German [22], Hungarian [23], and French [21]. Prior to deeming the translated FNS as a suitable tool for measuring food neophobia, it was essential to conduct validation studies evaluating its characteristics. Additionally, alterations, such as modifications and deletions of certain words or questions, were made across all translations to enhance the scale’s comprehensiveness and adaptability to diverse cultural contexts [5,12]. 

The objective of this study was to translate the FNS questionnaire into Romanian, facilitate its cultural adaptation for the Romanian population, and assess its validity using a national study group, while also comparing the results with those from various other countries.

## 2. Materials and Methods

### 2.1. Questionnaire

The translation of the FNS questionnaire into Romanian was conducted by a dietician and public health professional, with subsequent editing by a native English speaker to ensure accuracy and clarity. The translation and validation process of the questionnaire in Romanian followed a multi-stage approach. This study used a digital form as a data collection instrument. This instrument was formatted and administered by Google^®^ Forms Platform (Google Inc., Mountain View, CA, USA). Its distribution took place directly among the participants through an electronic link with digital monitoring by the authors. An introductory statement outlining the study’s purpose and the assurance of data anonymity preceded the initial query to obtain informed consent. Participants indicated their agreement to use their responses for the study by clicking “I agree”. The questionnaire consisted of two sections: the first comprised the original 10 items of the FNS, while the second included 7 questions related to socio-demographic factors such as age, gender, health status, pregnancy, educational level, and rural/urban residence. The 10 items of the FNS were retained in their original form as developed by Pliner and Hobden [8] and were translated into Romanian. Terminology adjustments were made in the questionnaire, replacing “ethnic” food and restaurants with “foreign” food and restaurants, aligning with the more prevalent and comprehensible usage in Romania. Respondents were instructed to rate each statement on a Likert scale ranging from 1 to 7, where 1 represented “strongly disagree”, and 7 denoted “strongly agree”. However, for questions 1, 4, 6, 9, and 10, the results were reversed, with “strongly agree” being assigned a value of 1 and “strongly disagree” being assigned a value of 7. The adopted methodology was first described by Pliner et al. [8] and used in several literature research studies [9,16,17,18]. A higher mean score, calculated by summing individual item scores on a Likert scale, indicates a decreased inclination to try new or unfamiliar foods (high in food neophobia, which we refer to as “neophobia”). Conversely, a lower mean score reflects a heightened willingness to explore novel foods (low in food neophobia, which we refer to as “neophilia”) [8]. 

### 2.2. Study Population 

The translated version underwent a pilot test in November 2023 involving 59 students in the medical field from two cities in the western part of Romania. The study was conducted in compliance with the Declaration of Helsinki. Approval for the research protocol was obtained from both the Ethics Committee of the “Victor Babes” University of Medicine and Pharmacy (no. 60/12 November 2018) and the hospital’s Ethics Committee (no. 3392/24 February 2023). Informed consent was obtained through a positive response to the initial question in the questionnaire, ensuring the non-collection of personally identifiable information. The distribution was facilitated through the Google Forms platform, with only 2 students refusing to answer the questionnaire.

Following the initial validation to confirm translation accuracy and term comprehension, the questionnaire was subsequently disseminated nationwide using the same Google Forms platform. The data collection period spanned from December 2023 to February 2024. Respondents were provided with an explanation of the questionnaire’s objectives and requested consent for the processing of their data within the study. Sample size for frequency in a population was calculated with OpenEpi [24], version 3, open-source calculator—SSPropor, based on data updated on 16 July 2023, using the most recent estimates from the Population Division of the Department of Economic and Social Affairs of the United Nations for July 2023–July 2024, with specific relevance to Romania [25].

Population size (for finite population correction factor or fpc) (N): 19,893,912
Hypothesized % frequency of outcome factor in the population (*p*): 50% ± 5
Confidence limits as % of 100(absolute ± %) (d): 5%
Design effect (for cluster surveys-DEFF): 1
Sample Size (n) for Various Confidence Levels
Confidence Level (%) 95% Sample Size: 385
Confidence Level (%) 90% Sample Size: 271

Equation Sample size n = [DEFF × Np(1 − *p*)]/[(d2/Z21) − α/2 × (N − 1) + *p* × (1 − *p*)]

The national sample comprised 375 individuals, providing a 90% confidence level and a 10% margin of error for the broader population of Romania. Following validation of the responses and exclusion of missing data, 359 respondents remained. A small percentage of answers had to be eliminated because the respondents either did not answer all the questions or, although they provided responses, did not give consent for the interpretation of their data. Notably, no personal data were collected during the study, ensuring participant confidentiality and adherence to ethical standards.

### 2.3. Validation of Questionnaire and Data 

The standard deviation, confidence level (α = 0.05), and skewness of each item in the questionnaire were computed using Microsoft Office 2013 Excel tools, with confidence intervals calculated for each item, considering skewness and an alpha value of 0.05. Once the results were centralized by summing the scores for each item, we applied the approach utilized in prior research to interpret the data. The possible range of scores theoretically fell between 10 and 70, where higher scores indicated greater levels of FN. Subjects were categorized into neophobia (high food neophobia) and neophilia (low food neophobia) groups based on their FNS results, with scores above 35 indicating neophobia and scores below 25 indicating neophilia [8]. Notably, individuals scoring between 25 and 35 were classified as exhibiting a neutral inclination, reflecting neither a strong aversion nor a heightened attraction to novel foods. 

The most commonly employed metric for assessing reliability, particularly internal consistency reliability, in research is coefficient alpha [26]. The questionnaire’s reliability was validated using the Cronbach’s alpha test.

$$\alpha =\frac{K}{\left(k-1\right)}\times \frac{{S_{y}}^{2}-Sum\;{S_{i}}^{2}}{{S_{y}}^{2}}$$

*α* represents the reliability coefficient (Cronbach’s alpha).
*K* denotes the number of items in the questionnaire.
*k* represents the average correlation between the items within the questionnaire.
*S_y_*^2^ represents the overall variance of the scale’s scores.
Sum *S_i_*^2^ represents the total of the scores for each individual item.

The Cronbach’s alpha coefficient serves as the preeminent test for evaluating the reliability of a translated instrument. This coefficient ranges from 0 to 1, with generally accepted α-values of 0.70 or higher being deemed satisfactory [27]. The robustness of the questionnaire was assessed using Spearman’s rho coefficient for correlation analysis of NG results at the FNS. These tests were conducted using Wessa, P. (2024), Free Statistics Software, Office for Research Development and Education, version 1.2.1 [28]. We interpreted the correlation coefficient using descriptors such as “weak”, “moderate”, or “strong” relationship [29].

### 2.4. Statistical Analyses

After the process of data gathering, the inputted values underwent analysis. The data of the subjects who refused consent or failed to respond to all the questions were excluded. The accepted data were statistically analyzed using Open Source Epidemiologic Statistics for Public Health. The software was last updated on 6 April 2013 and accessed on 30 March 2024. An analysis was conducted on the distribution of respondents into categories characterized by varying levels of food neophobia. Additionally, an analysis was conducted to examine the association between gender, health status, and place of residence (rural/urban) of the respondents with their classification into the more neophobic category based on their scores obtained from the FNS. The ANOVA test conducted included measures such as Sum of Squares, Degrees of Freedom, Mean Square, F-Statistics *p*-value, Chi-Square *p*-value.

### 2.5. Use of OpenAI ChatGPT-3.5 

ChatGPT-3.5 [30] (GPT-3.5, OpenAI’s large-scale language-generation model) was employed for language and grammar checks within the article. The authors carefully reviewed, edited, and revised the ChatGPT-generated texts to their own preferences, assuming ultimate responsibility for the content of the publication.

## 3. Results

The mean age of the 359 respondents from the national group was 38.07 years, with a standard deviation of 10.75 and a female-to-male sex ratio of 4:1 (Table 1).

A methodical approach was employed for the questionnaire translation. We selected the form in the Romanian language that most accurately and clearly conveys the meaning of the original FNS questionnaire [8] for each question (Supplementary Table S1). 

As shown in Table 2, both the pilot group (PG) and the national group (NG) exhibited a similar trend in the standard deviation and mean scores for each question. This trend was also observed in the skewness of the values obtained for each question in both PG and NG.

The confidence coefficients obtained are consistent with the Cronbach’s alpha coefficient. PG’s coefficient shows a high degree of correlation with a Cronbach alpha of 0.803 (95%CI%: 0.772, 0.831), while NG’s Cronbach’s alpha coefficient is 0.837 (95%CI: 0.811, 0.861).

Table 2 provides a comprehensive overview of the descriptive statistics associated with each item on the FNS. The mean FNS score for the national group is 31.86. Categorizing neophilia as an FNS score less than 25 and neophobia as an FNS score over 35, it is evident that the majority of respondents (69.20%) fall within the neutral group, with an FNS score between 25 and 35. Furthermore, the proportions of individuals categorized as low in food neophobia and neophobes are distinct, standing at 12.59% and 18.21%, respectively. For the pilot group, the percentage of neophobic respondents was found to be 42.10%, and neophilic respondents were categorized in 10.52%.

**Figure 1:**
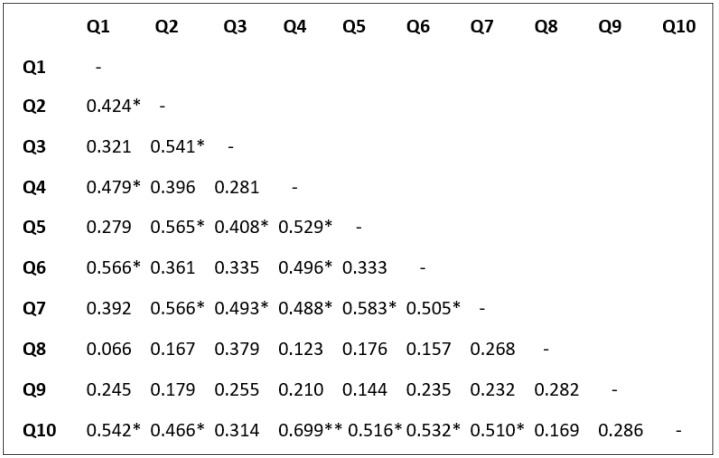
Correlations for all pairs of data series by Spearman method. *: *p* < 0.005; **: *p* < 0.001.

The findings derived from the Spearman correlation test, presented in Figure 1, indicate that 46.6% of the correlations exhibit a moderate to strong relationship.

As seen in Table 3, there were no significant differences in FNS score across the National Group, for most factors except for educational level, where the difference is borderline significant (*p* = 0.078).

## 4. Discussion

This work signifies a pioneering effort in Romania, involving the translation and validation of the Food Neophobia Scale while concurrently assessing neophobia within the broader population.

In the field of food neophobia assessment, the Food Neophobia Scale is recognized as a valuable tool for evaluating individuals’ hesitancy or resistance toward trying new or unfamiliar foods. When selecting the FNS, we considered other existing instruments that analyze food behaviors in children and adults. Additionally, we emphasize the distinctiveness of the FNS compared to these instruments.

The Children’s Eating Behavior Questionnaire, designed to explore various eating behaviors in children, covers factors such as responsiveness to and enjoyment of food, satiety responsiveness, slowness in eating, fussiness, emotional overeating, emotional undereating, and desire for drinks. While the CEBQ encompasses a broad spectrum of eating behaviors, its focus on early precursors of obesity may not align with the specificity required for assessing food neophobia. In contrast, the FNS, explicitly tailored to measure the trait of food neophobia, provides a more targeted and nuanced evaluation in this particular domain [31].

Similarly, the Fruit and Vegetable Neophobia Inventory evaluates neophobia related to vegetables and fruits but may encounter issues with answer transmission between its two subscales, potentially leading to response bias. The FVNI’s specific focus on fruits and vegetables limits its applicability to a broader assessment of food neophobia across various categories, a strength exhibited by the more generalized FNS, which comprises new and different foods and foreign cuisine [32].

The Food and Eating Questionnaire, exploring attitudes toward food through a preference test, shows correlations between neophobia scores and liking or willingness to try foods. However, the FNS diverges in its correlation with FEQ, emphasizing the need to consider different dimensions and nuances captured by each instrument. Notably, the FNS’s general focus on reluctance to try new foods without specific consideration of sensory aspects distinguishes it from the FEQ [33].

The FNS, when applied together with other scales such as the General Neophobia Scale (GNS) and Food Technology Neophobia Scale (FTNS) [34], offers a multi-dimensional approach. While the FTNS measures neophobia specifically in the context of technology, it does not consider all factors influencing neophobia. On the other hand, the GNS captures general neophobia, but it may also reflect personality traits associated with the fear of new situations rather than food-related behaviors. Nevertheless, the correlations observed between these two scales and the FNS offer insights into whether neophobic tendencies in individuals are generalized or specific to certain types of foods. This underscores the importance of carefully selecting or modifying assessment tools based on research objectives and the dimensions of neophobia being explored [18].

Furthermore, the Variety Seeking Tendency Scale (VARSEEK) [35] and the teacher-administered Taste-Test Tool (TTT) [36] provide alternative perspectives on variety seeking and willingness to try specific foods, respectively. VARSEEK, despite its time efficiency by only having eight items, may lack relevance due to outdated terminology. The TTT, although practical for assessing children’s attitudes at the classroom level, concentrates on specific food categories and may not capture the comprehensive nature of neophobic traits like the FNS.

The growing interest in multicultural cuisines worldwide in recent decades has prompted the expansion of evaluation tools to include items addressing this area. The Food Situation Questionnaire (FSQ) introduces cultural situations but may suffer from potential biases in assessing children’s approaches to foods. The FNS, with its broader focus on general reluctance or aversion to trying new or unfamiliar foods, complements the FSQ, offering a more comprehensive evaluation of neophobic tendencies.

Concerns about the universal validity of instruments like the Food Neophobia Questionnaire (FNQ) [37] for French children or the Food Neophobia Scale for Children (FNSC) highlight the importance of considering cultural and temporal relevance [38]. The WillTry Instrument, while comprehensive, may prove time-consuming in comparison to the FNS, emphasizing the trade-off between efficiency and comprehensiveness [39].

As such, the FNS emerges as a robust and targeted instrument for assessing food neophobia, offering a nuanced perspective that complements or surpasses the capabilities of other scales. Its focused approach, capturing general reluctance or aversion to trying new or unfamiliar foods, positions the FNS as a valuable tool deserving validation and translation into diverse languages, including Romanian, to facilitate its application in various academic studies. Researchers should weigh the specific dimensions they intend to explore, considering the cultural and temporal contexts, when selecting an instrument for their studies.

Likert scales, particularly the seven-point scale employed in the FNS, offer a nuanced approach to grading responses during the validation process. It is important to note that the level of detail in responses is significantly more extensive in seven- and nine-point scales than in their three- and five-point counterparts. Despite the inherent ease and efficiency of three- to five-point scales, the deliberate selection of a seven-point Likert scale for FNS reflects a strategic decision aimed at achieving heightened reliability during the validation phase. This deliberate choice not only aligns with the practical advantages of shorter scales but also emphasizes the paramount importance of enhanced reliability in accurately capturing the subtleties of respondents’ attitudes towards food neophobia [40].

When evaluating the internal consistency of the FNS across diverse language adaptations in this study, the Romanian-translated FNS stood out with a Cronbach’s alpha of 0.84. In contrast, the Brazilian Portuguese version scored 0.916 [16]. These findings highlight a substantial distinction between the Romanian version and its counterparts in various languages, as evidenced by lower alpha values observed in other translations such as the German version scoring 0.79, the French 0.82 [22], and the Spanish scoring 0.82 [20]. This robust internal consistency further underscores the reliability of the Romanian adaptation of the FNS, reinforcing its suitability for research applications within the Romanian population.

The questionnaire’s robustness was validated by the strong correlation between Q4 and Q10. It is logical for respondents to prefer restaurants offering international specialties if they exhibit an interest in food from other nations. Similarly, the correlation between Q2 and Q3 is expected; if a respondent lacks confidence in trying new foods, they are likely to avoid meals with unfamiliar ingredients. This trend extends to the relationship between Q2 and Q5; skepticism towards new foods may lead to a perception of meals with foreign ingredients as unusual.

The association between Q1 and Q2 is less understandable; it is difficult to explain how a person’s food behavior may be consistent when they are curious about new types yet do not trust them. Except for the pair Q1 and Q9, the responses to food acceptance questions (Q1, Q4, Q6, Q9, and Q10) align with expectations. Respondents who are open to new variations may exhibit selective eating habits, which is natural. Conversely, questions probing the rejection of new foods (Q2, Q3, Q5, Q7, and Q8) are generally correlated, except for Q2 and Q3 with Q8, which were expected to correlate strongly.

Individuals wary of new food types and ingredients tend to express caution about their food choices. These discrepancies in responses may partly stem from limitations in the online questionnaire’s ability to capture nuanced details. 

As “neophobia” has already been discussed in the Introduction, “neophilia” demands further details. The term “neophilic” denotes a distinctive attribute wherein individuals or groups manifest a pronounced proclivity and readiness to engage in the exploration and experimentation of novel and unfamiliar foods [7,41]. This characteristic is indicative of a heightened receptiveness towards embracing culinary diversity and a willingness to venture beyond familiar dietary choices. In the context of gastronomic preferences, a neophilic disposition indicates a strong inclination and curiosity toward expanding one’s palate, promoting culinary exploration beyond conventional or routine dietary patterns. Such an inclination toward embracing food novelty not only reflects an individual’s gastronomic openness but also holds implications for broader discussions in the realms of nutrition, cultural studies, and consumer behavior. The neophilic trait thus becomes a pertinent focal point for academic inquiry, offering insights into the dynamic interplay between personal preferences, societal influences, and the evolving landscape of dietary choices [10,21,42]. In examining respondents’ attitudes towards a constant sampling of new and different foods, the Romanian study population (mean 2.69) showed a higher tendency to curiosity compared to the original FNS (3.84) [8], as well as to Korean (4.3) [9] and Brazilian Portuguese (2.9) [16] populations. This variance can be partly attributed to temporal factors, considering the original FNS scale was proposed in 1992, while the Korean cohort was analyzed in 2011, and the Brazilian in 2015. 

Regarding food exploration, similar patterns emerge. Participants display a moderate inclination towards culinary novelty, as evidenced by an average score of 3.38 on the statement “I don’t trust new foods”. This suggests a lower level of caution and skepticism when encountering unfamiliar food items compared to other cohorts (original FNS: 2.98, Korean: 3.2, Brazilian: 2.4) [8,9,16]. 

Furthermore, when assessing respondents’ aversion to ambiguity in food content, it becomes apparent that individuals are less reserved about consuming food with undisclosed ingredients, as reflected in the moderately high mean score of 3.90 on the statement “If I don’t know what is in a food, I won’t try it”. All the three previously compared studies show a lower mean (original FNS: 3.61, Korean: 3.4, Brazilian: 3.2), indicating a higher reservation with unknown food content [8,9,16].

Moreover, respondents exhibit a relatively robust openness towards food diversity in terms of intercultural dining preferences, with a low mean score of 2.53 on the statement “I like foods from different countries”, compared to the original FNS (3.61) and Korean (3) [8,9]. The Brazilian cohort reveals a lower mean score of 2.1, suggesting a trend towards embracing culinary diversity and experiencing a wide array of international cuisines [16].

“Ethnic foods” typically refer to cuisine characteristic of a particular cultural or regional group, often featuring unique ingredients, flavors, and cooking techniques associated with that group’s heritage or traditions. In terms of the appearance of ethnic foods, Romanian respondents exhibit reluctance towards “weird-looking foods” with a mean score of 2.74, as with the original FNS (2.57) and the Brazilians (2.4), but contrasting with Koreans and Spaniards, who show higher openness with a mean value of over 3.5 [8,9,16,20]. Notably, in some cultures, familiarity plays a crucial role in the inclination to consume unconventional food items like insects [3,5].

In the context of Question 6, designed as a reverse question, it is observed that the Romanian cohort exhibits a comparable mean Food Neophobia Scale (FNS) score to both the Brazilian [16] and Korean [9] counterparts, registering within the range of 2.3 to 2.7. This finding implies a moderate inclination to try new food at dinner parties. Conversely, the Hungarian [23] demographic displays a notably higher mean FNS score of 4.08. This suggests a prevailing tendency among Hungarian individuals to refrain from sampling novel food items during communal dining events, thus highlighting a discernible divergence in culinary attitudes and behaviors.

Regarding the feeling of being afraid to eat foods never tried before, Romanian respondents described a moderate fear in agreement with the previously described cohorts, with a mean score of 2.92 for this item [8,9,16,20]. The Hungarian study conducted more recently has a neophilic tendency with a mean score of 3.68 [23]. Feeling afraid to eat things you have never had before is a common response, often stemming from uncertainty about the taste, texture, or potential adverse reactions. It is natural to have a preference for familiar foods because they provide a sense of comfort and predictability. Trying new foods can feel like stepping into the unknown, which can be intimidating. Additionally, concerns about food allergies, digestive issues, and several comorbidities may contribute to this fear. Overall, it is a cautious approach to safeguard against potential discomfort or unpleasant experiences. 

Remarkably, the Romanian population exhibited the highest mean value (5.07) for statement 8: “I am very particular about the foods I will eat”. In contrast, the original FNS yielded a mean value of 3.74 [8], while the Brazilian population scored 2.1 [16] and Koreans 4 [9]. The Hungarian population also demonstrated a noteworthy mean value of 3.94 [23] for this statement. These variations suggest diverse inclinations among populations regarding adherence to specific dietary preferences and habits, potentially influenced by taste preferences, health considerations, or cultural norms. The observed preference for selectivity underscores the importance of individual discernment and personal criteria in shaping dietary decisions. Additionally, agreement with statement 8 consistently indicates avoidance behavior towards certain foods. 

Tuorila et al. suggested that item 8 may be indicative of a heightened interest driven by dietary restrictions rather than directly stemming from food neophobia [5]. Conversely, Ritchey et al. suggested that item 8 is more closely associated with specific health concerns [12]. Koivisto and Sjödén highlighted that item 8 may not distinctly capture food neophobia [13]. Consequently, relying solely on the isolated evaluation of item 8 may not suffice for assessing food neophobia but could serve better for evaluating healthcare-related aspects. 

The Romanian population displays a moderate inclination towards trying new foods, scoring 3.55 for the statement “I will eat almost everything”, slightly below the original FNS mean of 4 for the same statement. Conversely, the Brazilian population shows a lower agreement level with a mean score of 2.4, indicating a higher selectivity. Koreans fall in the middle with a mean score of 3, demonstrating a moderate willingness to try new foods. Similarly, Hungarians also exhibit a moderate openness to new foods, scoring 3.18 for the statement. These variations underline the cultural influences on individuals’ food preferences and willingness to experiment. However, concerns have been raised about the accuracy of item 9 in several studies. Notably, Ritchey et al. argue that its broad nature may lead to misinterpretation. For example, vegetarians might be open to trying new foods but would not eat “anything” due to dietary restrictions. Hence, item 9 on its own fails to accurately measure food neophobia [12]. Similarly, Zhao et al. [15] suggest that item 9, along with item 8, primarily assesses habitual picky eating behaviors rather than food neophobia. Guidetti et al. [1] further note that item 9, along with items 3 and 8, fails to distinguish between food neophobia and other dietary preferences. The problem with item 9 is evident from its low item–whole correlation in the original study of Pliner and Hobden [8]. Thus, Ritchey et al. [12] advocate for careful consideration of this item, suggesting its exclusion from analysis if it does not align well with the overall scale.

Delimitating food neophobia from other eating or nutritional disorders constitutes a fundamental endeavor within the realms of nutritional psychology and clinical practice. In the developmental phase of childhood, it is not uncommon to observe instances of selective eating habits, commonly referred to as picky eating, alongside occurrences of food neophobia. These behavioral patterns, inherent to childhood, often persist and exert influence over eating behaviors in adulthood, thereby underscoring their significance as predictive factors in the continuum of dietary practices across the lifespan [43,44,45].

Conversely, eating and nutritional disorders encompass a broader spectrum of conditions, including but not limited to anorexia nervosa, bulimia nervosa, binge eating disorder, and orthorexia nervosa, each marked by intricate psychological and behavioral patterns related to food consumption, body image, and weight management [46,47].

Food neophobia has been correlated with a decrease in dietary diversity, leading to unfavorable health outcomes, notably an elevated susceptibility to type 2 diabetes and an increased body mass index [48,49]. 

The process of delineating food neophobia from other eating or nutritional disorders necessitates meticulous examination of several factors, notably encompassing individuals’ attitudes towards food, dietary habits, nutritional intake, and psychological well-being. This differentiation relies upon diagnostic criteria, the utilization of standardized assessment methodologies, and clinical discernment.

A nuanced understanding of the distinctions between food neophobia and other eating or nutritional disorders holds paramount significance. Such comprehension underpins accurate diagnostic practices, facilitates the formulation of tailored treatment modalities, and informs the development of targeted intervention strategies designed to address the unique needs and challenges presented by each individual.

The mean scores for the reverse question “I like to try restaurants with foreign cuisine” provide insights into attitudes towards culinary exploration across different populations. Among Romanians, the mean score of 2.53 indicates a tendency towards less enjoyment of trying restaurants with foreign cuisine. This suggests a preference for familiar Romanian cuisine over exploring foreign culinary experiences.

In contrast, the original FNS [8], demonstrates a lesser enjoyment of trying restaurants with foreign cuisine, with a mean score of 3.69. This reflects a cultural environment where there is significant appreciation for diverse cuisines and a willingness to explore new culinary experiences.

Similarly, Koreans [9] also show a relatively high mean score of 3.4, indicating a comparable level of enjoyment in trying restaurants with foreign cuisine as the original FNS. This suggests a similar level of openness and curiosity about exploring diverse culinary options.

Meanwhile, Brazilians exhibit a lower mean score of 2.2, indicating a lesser enjoyment of trying restaurants with foreign cuisine. This preference for familiar Brazilian cuisine over foreign culinary experiences could be influenced by Brazil’s rich culinary tradition [16].

However, the significantly higher mean score for Hungarians, at 4.70, suggests a strong enjoyment of trying restaurants with foreign cuisine. This indicates a pronounced openness and enthusiasm for exploring diverse culinary offerings among Hungarians, potentially driven by the curiosity about different cultures and cuisines that has developed rather in this last decade [23].

These variations highlight the diversity of food preferences and attitudes towards culinary exploration among populations, influenced by cultural, economic, temporal, and social factors.

Examining the FNS scores across various populations reveals notable variations. For example, the Romanian national population exhibits a mean FNS value of 31.86, with a standard deviation (SD) of 9.78. In contrast, the Hungarian population shows a slightly higher mean FNS value of 39.75 (SD = 12.599) [12]. Similarly, a representative sample of Finnish adults has an FNS value of 38.0 (SD = 10.5) [50], showcasing a higher level of food neophobia than observed in Romania. Other studies further accentuate this pattern, with notably lower FNS values reported, such as 31.74 (SD = 10.98) for Spanish adults [20]. The FNS value for Korean adults aged 20–40 years is 33.5 (SD = 9.0) [12], slightly higher than the Romanian mean [9]. This comprehensive comparison indicates that, on average, Romanian adults exhibited a lower FNS value compared to their diverse international counterparts, with the exception of Spain. 

Examining the tendency for food neophobia within student cohorts, the pilot group of Romanian students showed a mean value of 28.2, with a standard deviation of 8.01. In contrast, Canadian university students reported an FNS value of 34.51 (SD = 11.86) [8], while their counterparts in the USA and Lebanon demonstrated a range from 29.8 (SD = 11.7) to 36.4 (SD = 9.8), respectively [11]. Moreover, Brazilian university students revealed an FNS value of 27.5 (SD = 11.1) [16]. Chinese college students scored 33.59 (SD = 8.14) [15]. This collective comparison indicates nuanced variations in food neophobia levels across different student populations, with Romanian students, on average, displaying a lower tendency toward food neophobia compared to the aforementioned countries, with the exception of Brazil.

Our findings align with some reports in the existing literature, affirming that the majority of people a neutral (69.20%) regarding the acceptance or curiosity to trying new foods, and a significant proportion are neophobes (18.21%) outnumbering neophiles (12.59%). This suggests that Romanian respondents exhibit a spectrum of attitudes towards novel foods, with a notable prevalence of neutrality and a substantial number displaying aversion or reluctance towards unfamiliar food items. In the study involving Brazilian consumers [12], similar percentages of all categories were observed: neophiles (10%), neutrals (72.5%), and neophobes (17.5%) compared to the Romanian population. A slight difference was noted in the Lebanese survey by Olabi et al. [11], with neophiles (7.9%), neutrals (70.6%), and neophobes (21.5%). Therefore, the average food neophobia score among Lebanese university students is higher than that of the Romanian population. However, several studies have reported comparable ratios among neophobes and neophiles. For example, a study conducted by a Hungarian research team on an adult population in 2020 has shown a slightly different distribution than ours, with 65.8% of respondents belonging to the neutral group, and the proportions of the neophile and neophobe groups nearly identical, at 17.4% and 16.8%, respectively. These findings highlight the coexistence of both fear of the new and a propensity for seeking out novelties among respondents [14]. Similar ratios were found by the Italian team led by D’Antuono and Bignami (neophile 17%, neutral 66%, neophobe 17%) [10], Tuorila et al. in Finland (17%, 66%, 17%, respectively) [5], and by Korean researchers Choe and Cho (15.6%, 71.4%, 13%, respectively) [9]. 

The present results have to be viewed in light of several study limitations. Although the study is numerically representative for the Romanian population, the data presented cannot be extended to the entire population because the sample obtained is not demographically representative. Therefore, the incidence of neophobia is influenced by this sampling bias. Another limitation of the study is that it was administered via an online application and not by trained investigators. A small percentage of answers had to be eliminated because the respondents did not answer all the questions or, although they answered, they did not give their consent for the interpretation of their data. Additionally, there was a notable difference in the proportion of men and women in the questionnaire sample. While efforts were made to ensure internal consistency, this gender disparity may have introduced bias and affected the representativeness of our findings. Future research should aim for a more balanced representation of genders to mitigate this potential limitation. 

## 5. Conclusions

Ultimately, the Romanian adaptation of the Food Neophobia Scale offers a fresh perspective for studies investigating the dietary habits of the population. This is particularly significant given the relatively limited awareness of food neophobia among local experts in the fields of nutrition and food science and technology in Romania. The translation of the neophobia investigation scale into Romanian contributes to the current research landscape by providing validated tools for studying eating behaviors, which are globally prevalent. Moreover, the translation of the FNS questionnaire into Romanian enhances its universal applicability, facilitating its utilization in multicentric studies across various countries. Despite its limitations related to the representativeness within the Romanian population, this study provides initial insights into food neophobia in Romania. These findings highlight a notable coexistence of both reluctance toward the unfamiliar and eagerness to explore novelties among the respondents. The discernible presence of both neophilic and neophobic tendencies within the surveyed population adds complexity to the interpretation of their food preferences and attitudes toward culinary exploration. The analysis of food neophobia stands to benefit researchers, healthcare professionals, the food industry, public health organizations, and consumers alike by providing insights into dietary behaviors and facilitating the development of interventions, products, and public health initiatives aimed at promoting diverse and nutritious eating habits. Subsequent investigations may undertake an examination of food neophobia across diverse pathologies, such as autism, diabetes, and anorexia.

## Figures and Tables

**Table 1:** Statistical description of the National Group.

Feature		Percentage
Gender	Male	20.61%
	Female	79.39%
Pregnant	Declared	4.21%
Age group	15–24	11.14%
	25–34	27.30%
	35–44	35.38%
	45–54	18.38%
	55–64	5.29%
	Over 65	2.51%
Health status	With chronic diseases	21.21%
	Without chronic diseases	78.79%
Residence area	Urban	76.88%
	Rural	23.12%
Educational level	University degree	82.45%
	High school	12.81%
	At least 8 classes	4.74%

**Table 2:** Statistical comparison for each item on FNS between Pilot Group (PG) and National Group (NG).

Statement	Mean		Standard Deviation		Skewness		Cronbach Alpha	
	NG	PG	NG	PG	NG	PG	NG	PG
1. I am constantly sampling new and different foods. (R)	2.69	2.29	1.39	1.15	0.88	1.02	0.82	0.78
2. I don’t trust new foods.	3.38	3.02	1.60	1.34	0.36	0.86	0.81	0.78
3. If I don’t know what is in a food, I won’t try it.	3.90	3.45	1.86	1.86	0.08	0.44	0.82	0.77
4. I like foods from different countries. (R)	2.53	1.95	1.32	1.09	1.15	1.17	0.82	0.79
5. Ethnic food looks too weird to eat.	2.74	2.02	1.52	0.98	0.92	1.52	0.82	0.78
6. At dinner parties, I will try a new food. (R)	2.55	2.32	1.26	0.90	1.19	0.72	0.82	0.79
7. I am afraid to eat things I have never had before.	2.92	2.43	1.58	1.29	0.78	1.08	0.81	0.76
8. I am very particular about the foods I will eat.	5.07	4.93	1.47	1.52	−0.86	−0.90	0.84	0.81
9. I will eat almost anything. (R)	3.55	3.71	1.83	1.76	0.44	0.10	0.85	0.80
10. I like to try restaurants with foreign cuisine. (R)	2.53	2.09	1.38	1.07	1.12	1.12	0.81	0.79

Items for which scoring is reversed are marked (R); NG, national group; PG, pilot group.

**Table 3:** ANOVA analysis among individuals with FNS > 35 based on demographic and comorbidity status.

	ANOVA					Test for Equality of Variance	
Source of Variation for FNS Score	Sum of Squares	d.f	Mean Square	*F* Statistics	*p*-Value	Chi Square	*p*-Value
Gender	80.2278	1	80.2278	2.26431	0.135057	0.136327	0.71196
No diseases/Chronic diseases	16.2058	1	16.2058	0.463263	0.497436	0.139119	0.709158
Rural/urban	9.92267	1	9.92267	0.275095	0.600918	3.37182	0.0663204
Educational level (1/2/3)	174.125	2	87.0626	2.60044	0.078528	13.9142	0.0009518
Age group	154.741	5	30.9483	0.84778	0.518824	17.8217	0.00317832

## Data Availability

The data presented in this study are available on request from the corresponding author due to ethical restrictions.

## Supplementary Materials

The following supporting information can be downloaded at: https://www.mdpi.com/article/10.3390/foods13091301/s1, Table S1: Food Neophobia scale English and Romanian version.
