# Supplementary material for: Exploring Adult Eating Behaviors and Food Neophobia: A National Study in Romania
Source: Foods. 2024 Apr 24;13(9):1301. doi: 10.3390/foods13091301 (PMC11083192; doi:10.3390/foods13091301)
Supplement: Supplementary file 1 [file foods-13-01301-s001.zip › foods-2967941-supplementary.pdf]

Supplementary Table 1. Food Neophobia scale English and Romanian version**.**

**English statement version**	**Romanian statement version**
1. I am constantly sampling new and different foods.	1. Am mereu curiozitatea sa incerc alimente diferite si noi
2. I don't trust new foods.	2. Nu am incredere in noi sortimente de mancare
3. If I don't know what is in a food, I won't try it.	3. Daca nu cunosc ingredientele care sunt in mancare, nu o incerc
4. I like foods from different countries.	4. Imi plac mancarurile specifice altor tari
5. Ethnic food looks too weird to eat.	5. Mancarea cu specific strain arata prea ciudat ca sa o consum
6. At dinner parties, I will try a new food.	6. La petreceri incerc sortimente noi de mancare
7. I am afraid to eat things I have never had before.	7. Imi este frica sa mananc alimente pe care nu le-am mai incercat pana acum
8. I am very particular about the foods I will eat.	8. Sunt foarte atent cu privire la alimentele pe care le voi manca
9. I will eat almost anything	9. Mananc aproape orice
10. I like to try new ethnic restaurants.	10. Imi place sa incerc restaurante cu specific strain
